# Endovascular Treatment for Subclavian Artery Stenosis and Occlusion: A Single-Center Retrospective Study

**DOI:** 10.7759/cureus.44699

**Published:** 2023-09-05

**Authors:** Lam Van Nut, Pham Xuan Vinh, Nguyen Lam Vuong

**Affiliations:** 1 Department of Vascular Surgery, Cho Ray Hospital, Ho Chi Minh City, VNM; 2 Thoracic and Vascular Department, Thu Duc City Hospital, Ho Chi Minh City, VNM; 3 Department of Medical Statistics and Informatics, Faculty of Public Health, University of Medicine and Pharmacy at Ho Chi Minh City, Ho Chi Minh City, VNM

**Keywords:** patency, occlusion, stenosis, arteriosclerosis, endovascular treatment, subclavian artery

## Abstract

Background: Subclavian artery stenosis and occlusion are common arterial diseases in the upper extremities, with atherosclerosis being the main cause. Endovascular treatment has emerged as a promising alternative to open surgical repair, but data are limited. This study aimed to evaluate the safety and effectiveness of endovascular procedures in the treatment of subclavian artery lesions at a tertiary vascular center in Vietnam.

Methods: A retrospective analysis was conducted on patients who underwent endovascular treatment for symptomatic subclavian artery stenosis or occlusion between October 2013 and April 2022. Clinical characteristics, procedural details, short- and long-term outcomes, and patency rates were assessed.

Results: Twenty-five patients were included in the study, with a mean age of 56.8 years. The majority of patients had risk factors for atherosclerosis, and all presented with symptoms related to subclavian artery disease. The endovascular procedures were successful in 96% of cases, with a low complication rate of 8%. During a median follow-up of 43 months, the overall patency rate was 92% at three years.

Conclusion: Endovascular treatment of subclavian artery stenosis and occlusion is a safe and effective option, with excellent long-term patency rates. These findings support the use of percutaneous revascularization as the first-line therapy, particularly in experienced centers. Further studies with larger sample sizes and longer follow-up periods are needed to confirm these results.

## Introduction

The subclavian artery is a frequently affected site for occlusive and stenotic arterial conditions in the upper extremities. The steno-occlusive disease of the subclavian artery is found in approximately 2-4% of the general population [1,2] and this prevalence rises to 11-18% in individuals with atherosclerotic diseases, such as peripheral artery or coronary artery disease [2,3].

The primary cause of subclavian artery stenosis or occlusion is atherosclerosis. However, less common etiologies include Takayasu's arteritis, radiation-induced vascular disease, and impedance caused by nearby tumors [2,4-6]. While many patients with subclavian artery stenosis or occlusion are asymptomatic, some may experience severe symptoms, including subclavian steal syndrome, upper extremity ischemia, or myocardial ischemia in those with a coronary arterial bypass grafting utilizing an internal mammary artery [7-9].

Revascularization becomes essential for patients experiencing symptoms or complications to prevent upper extremity necrosis, stroke, and mortality. Traditionally, open surgical repair has been the main approach for addressing this issue. However, this major surgery involves challenging techniques and carries a high rate of complications [10,11]. Fortunately, recent advancements in materials and technical expertise have ushered in a new era, with endovascular intervention replacing surgery as the first-line treatment option [12,13]. Several studies have highlighted the feasibility and favorable outcomes of endovascular treatment for subclavian artery stenosis or occlusion [14-20]. However, these studies have been limited by their retrospective nature and small sample sizes, with no available randomized trials.

In Vietnam, endovascular intervention for subclavian artery stenosis or occlusion has been employed since 2013 in select tertiary vascular centers. While results are anticipated to be as promising as those reported in other countries worldwide, no specific data from Vietnam are currently available. With the present study, our objective is to evaluate the short- and long-term outcomes of endovascular procedures performed at our institution over a span of 10 years in treating subclavian artery lesions.

## Materials and methods

Study design and population

This study is a retrospective analysis of patients who underwent endovascular treatment for subclavian artery stenosis or occlusion at the Department of Vascular Surgery, Cho Ray Hospital, Ho Chi Minh City, Vietnam, between October 2013 and April 2022. Cho Ray Hospital is a renowned tertiary care center, serving as a high-volume facility for vascular treatment in Southern Vietnam, catering to a population of nearly 50 million people. Ethical approval for this study was obtained from the Ethics Committee of the University of Medicine and Pharmacy at Ho Chi Minh City (Approval No. 540/HĐĐĐ-ĐHYD, dated 2 June 2022).

We included all patients diagnosed with symptomatic stenosis or occlusion of the subclavian artery, suspected to be caused by atherosclerosis, and who subsequently underwent endovascular treatment. Patients with stenosis or occlusion due to other etiologies, such as trauma, extrinsic obstruction by tumors, or thrombosis leading to acute disease, were excluded from the study. Prior to performing the intervention, written informed consent was obtained from all patients and their relatives.

Revascularization for subclavian artery lesions in our institution followed the following standard criteria: (i) stenosis of 70% or above or complete occlusion of the artery's inner diameter, and (ii) corresponding symptoms with subclavian artery stenosis or occlusion, such as the presence of steal syndrome with vertebrobasilar insufficiency symptoms (e.g., vertigo, diplopia, or optometry) or chronic upper limb ischemia (e.g., arm claudication, rest pain, numbness, or ulcers). The indication for revascularization was determined on a case-by-case basis, considering individual patient factors, including symptomatology, concomitant medical conditions, surgical risk, available vascular access routes, life expectancy, and patient preferences.

Data collection

Data for this study was retrieved from medical records, radiology reports, and operative notes. Demographic information, such as age, sex, and comorbidities, was collected. Preoperative clinical symptoms and imaging findings were recorded. Intra- and postoperative parameters, as well as any complications, were also documented.

The endovascular intervention was carried out under either local or general anesthesia. During the procedure, systemic heparin was administered to maintain an activated clotting time of approximately 250 seconds. We preferred a retrograde approach to cross the subclavian lesion, utilizing an ipsilateral brachial approach. If the retrograde access was unsuccessful, an antegrade approach with femoral access was utilized as an alternative. Following the recanalization and balloon angioplasty of the subclavian disease segment, the procedure was completed by deploying a balloon-expandable stent.

Early results of the intervention were evaluated by intraoperative angiography after stenting, and brachiocephalic vessels duplex ultrasonography was conducted before patients were discharged from the hospital. Upon discharge, all patients were prescribed oral dual antiplatelet therapy, comprising acetylsalicylic acid (ASA) 75 mg and clopidogrel 75 mg daily for three months, followed by a single antiplatelet agent for lifelong therapy. Patients underwent follow-up appointments at 1, 3, 6, and 12 months after discharge, and subsequently on an annual basis at the outpatient department. During each visit, clinical examination and duplex ultrasound were performed. Computed tomographic angiography (CTA) was carried out if there were suspected cases of subclavian artery restenosis or occlusion. Reintervention was indicated in cases of restenosis of the subclavian artery of more than 70%, the presence of steal syndrome with vertebrobasilar insufficiency symptoms, and/or chronic upper limb ischemia.

Outcome measurement

Short-term outcomes were assessed based on technical success, postoperative complications, and 30-day mortality. Technical success was defined as the achievement of a patent subclavian artery without significant residual stenosis (less than 30% of the inner diameter) as determined by intraoperative angiography after stenting, and duplex ultrasound before the patient's discharge. Postoperative complications included any intraoperative accidents or occurrences of organ involvement that necessitated postoperative medical or interventional intervention or prolonged the length of hospital stay. The 30-day mortality category included all deaths from any cause within 30 days following the intervention.

Long-term outcomes encompassed the primary patency of the subclavian artery, occurrences of cardiac and neurologic events, as well as all-cause mortality during the follow-up period. The identification of restenosis was based on the recurrence of vessel lumen narrowing equal to or greater than 70%. Cardiac events were recorded if there were any incidents involving the heart, such as ventricular fibrillation, complete heart block, myocardial infarction, cardiac arrest, or cardiac death. Neurologic events included any incidents involving the brain, such as transient ischemic attack, ischemic stroke, intracranial bleeding, coma, or brain death. Any cardiac and neurologic events, as well as all-cause deaths, were documented based on medical records in the event of hospital admission or through the death certificate in case of mortality.

Statistical analysis

The statistical analysis included the summary of baseline characteristics and patient outcomes. Continuous variables with a normal distribution were presented as mean ± standard deviation, while those with a skewed distribution were summarized as median and interquartile range (IQR). Categorical variables were expressed as frequencies and percentages. For long-term outcomes, Kaplan-Meier estimates were utilized, and the results were depicted using Kaplan-Meier curves. The statistical analysis was performed using the R statistical software version 4.1.3 (R Foundation for Statistical Computing, Vienna, Austria).

## Results

During the study period from October 2013 to April 2022, a total of 25 patients with symptomatic subclavian artery stenosis or occlusion underwent endovascular treatment at our hospital. The characteristics of the patients are presented in Table 1. Male patients were predominant, accounting for 64% (16 out of 25 patients). The age of the patients ranged from 30 to 78 years, with a mean age of 56.8 years. The majority of patients had several risk factors for atherosclerosis, including hypertension (72%), dyslipidemia (68%), and smoking (64%). Additionally, 44% of patients had concomitant peripheral arterial disease in the lower extremities. Furthermore, 28% had concomitant carotid disease, and 4% had concomitant coronary arterial disease. All patients experienced symptoms related to subclavian stenosis or occlusion, with arm claudication being the most prevalent symptom (100%), followed by numbness (84%) and pulse loss (68%). Less common symptoms included cold skin (36%), ulcers (24%), vertigo (24%), pale skin (8%), and weakness (8%).

**Table 1:** Patient characteristics Summary statistics are n (%), mean ± standard deviation (min; max), and median (25th; 75th percentiles). CAD, coronary artery disease; PAD, peripheral arterial disease

	All patients (N=25)
Sex	
Male	16 (64.0)
Female	9 (36.0)
Age (years)	56.8 ± 14.2 (30; 78)
Comorbidities	
Hypertension	18 (72.0)
Dyslipidemia	17 (68.0)
Smoking	16 (64.0)
Lower limb PAD	11 (44.0)
Carotid stenosis	7 (28.0)
Diabetes	4 (16.0)
CAD	1 (4.0)
Previous stroke	1 (4.0)
Symptoms	
Arm claudication	25 (100.0)
Numbness	21 (84.0)
Pulse loss	17 (68.0)
Cold skin	9 (36.0)
Ulcer	6 (24.0)
Vertigo	6 (24.0)
Pale skin	2 (8.0)
Weakness	2 (8.0)
Length of follow-up (months)	43 (37; 48)

Table 2 displays the lesion characteristics as assessed by CTA. The majority of patients (76%) had the main lesion located in the left subclavian artery. Most of the lesions were restricted to the proximal segment only (76% of patients), while in 20% of patients, the lesions extended from the proximal to medial segment, and in 4% of patients, the lesion involved the entire subclavian artery. The severity of most lesions (64%) was evaluated as stenosis ranging from 70% to 95%, whereas 20% of lesions were assessed as occlusions.

**Table 2:** Lesion characteristics Summary statistics are n (%).

	All patients (N=25)
Side	
Left	19 (76.0)
Right	6 (24.0)
Lesion location	
Proximal only	19 (76.0)
Proximal to medial	5 (20.0)
Proximal to distal	1 (4.0)
Lesion severity	
Stenosis 70-95%	16 (64.0)
Stenosis 96-99%	4 (16.0)
Occlusion	5 (20.0)

Table 3 presents the procedural characteristics of the endovascular treatments. The majority of procedures (88%, 22 cases) were performed under local anesthesia, while in the remaining three patients (12%), the procedure was conducted under general anesthesia. The mean operating time for the procedures was 71.6 minutes, ranging from 40 to 120 minutes. In 20 patients (80%), successful retrograde access was achieved through the ipsilateral brachial artery. However, in the remaining five patients (20%), retrograde access was not feasible, and the antegrade approach through the femoral artery access was utilized. The lesion characteristics assessed intraoperatively were consistent with those assessed by CTA, with most lesions (76%, 19 cases) located in the proximal segment only, and the majority being classified as stenosis (76%, 19 cases).

**Table 3:** Procedural characteristics Summary statistics are n (%) and mean ± standard deviation (min; max).

	All patients (N=25)
Anesthesia	
Local anesthesia	22 (88.0)
General anesthesia	3 (12.0)
Operating time (mins)	71.6 ± 20.1 (40; 120)
Accessed site	
Ipsilateral brachial artery	20 (80.0)
Femoral artery	5 (20.0)
Lesion location	
Proximal only	19 (76.0)
Proximal to medial	4 (16.0)
Proximal to distal	2 (8.0)
Lesion severity	
Stenosis 70-95%	12 (48.0)
Stenosis 96-99%	7 (28.0)
Occlusion	6 (24.0)
Complications	2 (8.0)
Postoperative hospital length of stay (days)	3.0 ± 0.7 (2; 5)
Technical success	24 (96.0)

During the procedures, two complications occurred, both in the form of hematoma at the accessed site, specifically at the brachial artery. In both cases, puncture-focused compression was successfully performed, and the hematomas were resolved within 24 hours. Overall, technical success was achieved in 96% of procedures (24 out of 25). Only one procedure was considered a failure due to a residual stenosis of more than 30% as revealed by intraoperative angiography after stenting. In an attempt to address in-stent stenosis, balloon angioplasty was performed but proved unsuccessful. This particular lesion was an occlusion located at the whole subclavian artery. Despite the failure to completely address the stenosis, the clinical symptoms of this patient showed improvement postoperatively.

Table 4 illustrates the significant improvement in clinical symptoms right after treatment when compared with before treatment. The most notable improvements were observed in arm claudication (reduced from 100% before treatment to 24% after treatment), numbness (reduced from 84% to 24%), and pulse loss (decreased from 68% to 4%). Only weakness did not show immediate improvement before discharge and required more time for resolution.

**Table 4:** Symptoms before and after intervention Summary statistics are n (%).

	Before treatment (N=25)	After treatment (N=25)
Arm claudication	25 (100.0)	6 (24.0)
Numbness	21 (84.0)	6 (24.0)
Pulse loss	17 (68.0)	1 (4.0)
Cold skin	9 (36.0)	2 (8.0)
Ulcer	6 (24.0)	3 (12.0)
Vertigo	6 (24.0)	2 (8.0)
Pale skin	2 (8.0)	1 (4.0)
Weakness	2 (8.0)	2 (8.0)

**Figure 1:**
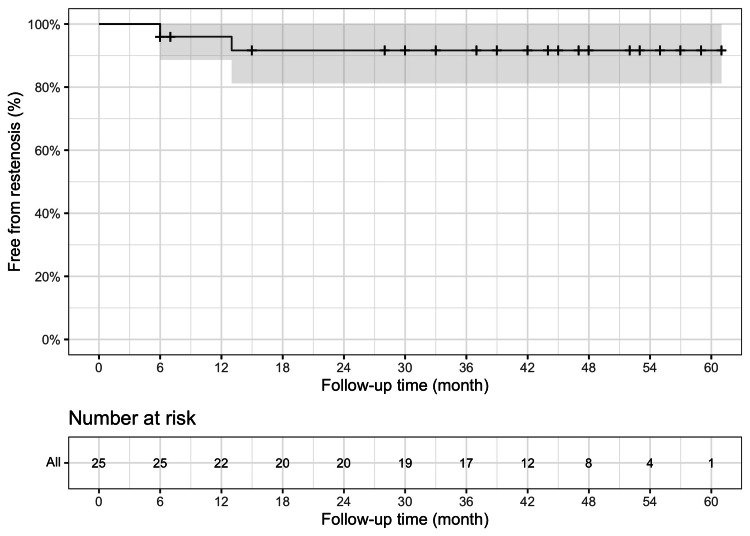
Kaplan-Meier curves for subclavian artery patency The line represents the Kaplan-Meier estimate, and the grey region represents the 95% confidence interval.

Over the median follow-up period of 43 months, there were no recorded cardiac or neurologic events, nor any deaths. However, restenosis of the subclavian artery was observed in two patients, one at six months and the other at 13 months after the intervention. Notably, the patient with restenosis detected at six months was the one with the technical failure described earlier. The Kaplan-Meier probability of freedom from restenosis was 96% at 1 year, 92% at 2 years, and 92% at three years (Figure 1).

## Discussion

The findings of this study highlight the safety and efficacy of endovascular treatment for subclavian artery diseases, with a low rate of minor complications (8%) and an overall excellent clinical success rate of 96%. The long-term patency rate, which remained above 90% after four years, further supports the positive outcomes of this intervention.

Percutaneous balloon angioplasty with stenting is a widely recognized approach for treating subclavian stenosis or occlusion. However, it appears to be less effective for occlusive lesions, as it has a lower success rate. In our study, the only technical failure case had an occlusive lesion that was located at the whole subclavian artery. Previous research demonstrated that the technical success rate was notably lower in patients with subclavian occlusions compared to those with stenosis lesions [17,21]. This is primarily attributed to the challenges posed by severe calcification and tortuosity of the subclavian arteries. Fortunately, the advancement of endovascular devices has provided new opportunities and indications for minimally invasive treatments in patients with occlusive diseases. While stent-supported angioplasty has been favored since the 1990s to avoid recoil and reduce the risk of recurrence, there is still a lack of randomized trials to definitively determine the superiority of stenting over plain angioplasty without stenting [22]. Some studies have shown potential advantages of stenting, while others reported no significant differences between the treatment strategies [23,24]. It is important to note that the decision to use stenting should consider factors such as lesion characteristics, the extent of calcification, and the presence of vascular dissections.

The overall complication rate observed in our study was low, with only two cases of minor hematomas at the accessed site. Importantly, no instances of embolization to the brain or limb were observed during the procedure. This finding aligns with previous literature, which consistently reports a low risk of cerebral or limb embolization, ranging from 0 to 5.7% [14,25,26]. While the number of embolic events remains small, access-site complications are more commonly encountered. As previously reported, using the brachial artery as the access site is associated with a higher incidence of complications compared to the femoral artery [27]. In our study, we also observed a significant increase in local adverse events when the brachial artery was used for access. An alternative option, the radial approach, which is commonly employed in coronary angiography, has been shown to have a lower access site complication rate. However, our department has limited experience with the radial approach in supra-aortic interventions thus far. For the majority of cases, femoral access with an antegrade procedure remains the preferred approach and is used as the primary option. Nevertheless, in certain cases, especially in the setting of total chronic occlusions, a combined approach with additional retrograde access can be crucial for achieving technical success. This approach allows for a double injection technique and the usage of guidewires in both directions, which can be advantageous in challenging cases.

During the follow-up period, our study demonstrated a low overall rate of restenosis, with a 92% patency rate at the three-year mark. These findings are in line with previous observational studies that reported primary patency rates ranging from 70% to 90% after two years [14,25,27]. Importantly, these patency rates are comparable to those reported after surgical interventions, reaffirming the effectiveness and durability of endovascular therapy as an alternative to surgery for subclavian artery diseases. For instance, in a retrospective study involving 110 patients with subclavian artery steno-occlusive disease treated by percutaneous angioplasty, the three-year patency rate was achieved in 93% of patients with subclavian stenoses and 65% of patients with occlusions [25].

Despite these positive findings, our study does have certain limitations. First, being a retrospective study conducted in a referral center, ensuring consistent follow-up for patients from different regions of Vietnam was challenging. Secondly, the sample size was relatively small, and there was no comparison group, mainly due to the low prevalence and incidence of symptomatic subclavian artery disease. As a result, future studies with larger sample sizes, longer follow-up durations, and comparison groups are essential and may provide additional data that could potentially influence or refine our current conclusions.

## Conclusions

Our retrospective single-center study has demonstrated that endovascular treatment for subclavian artery stenosis and occlusion is not only safe but also highly effective, yielding excellent long-term patency rates. These results are consistent with previous reports and add further evidence to support percutaneous revascularization as the preferred first-line therapy for these conditions. However, it is important to acknowledge that further studies with larger sample sizes and longer follow-up periods are needed to confirm and validate these findings.
